# Transmembrane and Juxtamembrane Structure of αL Integrin in Bicelles

**DOI:** 10.1371/journal.pone.0074281

**Published:** 2013-09-12

**Authors:** Wahyu Surya, Yan Li, Oscar Millet, Tammo Diercks, Jaume Torres

**Affiliations:** 1 School of Biological Sciences, Nanyang Technological University, Singapore, Singapore; 2 Structural Biology Unit, CIC BioGUNE, Derio Vizcaya, Spain; Lerner Research Institute, United States of America

## Abstract

The accepted model for the interaction of α and β integrins in the transmembrane (TM) domain is based on the pair αIIbβ3. This involves the so-called outer and inner membrane association clasps (OMC and IMC, respectively). In the α chain, the OMC involves a GxxxG-like motif, whereas in the IMC a conserved juxtamembrane GFFKR motif experiences a backbone reversal that partially fills the void generated by TM separation towards the cytoplasmic half. However, the GFFKR motif of several α integrin cytoplasmic tails in non-bicelle environments has been shown to adopt an α-helical structure that is not membrane-embedded and which was shown to bind a variety of cytoplasmic proteins. Thus it is not known if a membrane-embedded backbone reversal is a conserved structural feature in α integrins. We have studied the system αLβ2 because of its importance in leukocytes, where integrin deactivation is particularly important. Herein we show that the backbone reversal feature is not only present in αIIb but also in αL-TM when reconstituted in bicelles. Additionally, titration with β2 TM showed eight residues clustering along one side of αL-TM, forming a plausible interacting face with β2. The latter orientation is consistent with a previously predicted reported polar interaction between αL Ser-1071 and β2 Thr-686.

## Introduction

Integrins are heterodimeric type I transmembrane (TM) proteins formed by non-covalent association of an α and a β subunit. In humans, 18 α-chains can interact with 8 different β-chains to form 24 different α/β heterodimers [1]. Each subunit contains a large extracellular domain, a single membrane-spanning α-helix and a short cytoplasmic tail [2]. By spanning the membrane, integrins serve as a dynamic linkage between cytoplasm and extracellular space, transducing signals across the membrane to mediate various functions [3]. Information transduction in integrins is bidirectional, inside-out or outside-in. Inside-out signaling involves separation of integrin cytoplasmic tails and TM domain, and subsequent ectodomain conformational changes, which alter the affinity of integrins for extracellular ligands [4]–[6]. Deletions or mutations that are expected to destabilize α/β association of TM or cytoplasmic domains in the α and β subunits, have been shown to activate integrins [7]–[9]. Conversely, inactivation is achieved by enhancing heterodimerization, e.g., using intermolecular disulfide bonds; in fact disulfide scanning of the αIIbβ3 TM region revealed the TM heterodimer interface [10].

Two models of heterodimeric α/β TM interaction were proposed based on electron microscopy and a computational approach showing different crossing angles for ‘resting’ and ‘activated’ states [5], [11]. For the first model, interaction was mediated via a GxxxG-like motif located in both α and β TM chains. This proposed mode of interaction is reminiscent of that found previously for the homodimer glycophorin A (GpA) [12]. For the second proposed mode of interaction [11], [13], the β helix is rotated by approximately 90° from its orientation in the GpA-like model. This latter model has been validated for αIIbβ3 using activating mutations at the transmembrane domain [14], cysteine cross-linking of the full integrin [10], [15], and NMR studies of the αIIbβ3 transmembrane heterodimer structure in bicelles [16]. Thus, this is currently accepted to represent the interaction of the two TM domains of αIIb and β3 in the resting state.

This accepted model has been further refined in the case of αIIbβ3 [15]–[17]. Specifically, the two TMs are in close proximity at the extracellular half of the membrane, forming an outer membrane association clasp (OMC) that involves the GxxxG-like motif in αIIb, Gly-972xxxGly-976, and Gly-708 in β3. The TMs separate towards the cytoplasmic half, and the void is partially filled with an inner membrane association clasp (IMC), formed by a juxtamembrane backbone reversal in αIIb that participated by two phenylalanines, Phe-992 and Phe-993. The latter are part of a conserved α chain GFFKR motif. Lastly, a salt bridge between Arg-995 of αIIb and Asp-723 of β3 has also been identified [16]. At present, no other detailed structural model for other integrin TMs is available.

In contrast to the above model for the juxtamembrane backbone reversal, the GFFKR motif in several α integrins e.g., in αIIb, α1, αM, αL, αX or α4 [18]–[24], formed an α-helical structure that was not membrane-embedded. However, most of these reports included the cytoplasmic tail without the TM domain, although in some cases they were myristoylated and attached to detergent micelles. Other reports included the TM domain, but the structure was determined in the presence of organic solvents or in presence detergent micelles. Thus, it is possible that the failure to observe such reverse turn in the above examples was due either to the absence of the TM domain, or to the fact that detergent micelles or organic solvents do not provide the appropriate environment for such structural feature.

Several reports show binding of several cytosolic proteins to a region in the α chain that encompasses this conserved motif, e.g., serine-threonine phosphatase 2A (PP2A) [25], nischarin [26]–[28], calcium- and integrin- binding protein 1 (CIB1) [29]–[31], sharpin [32], or mammary-derived growth inhibitor (MDGI) [33]. Although the precise binding location of these proteins has not been elucidated, these data suggest that an α-helical solvent exposed structure is physiologically relevant.

Although the membrane-embedded reverse backbone has been observed in the presence of the TM domain and lipids, whether in bicelle systems or membranes [15], [16], it has only been reported for αIIb integrin. Therefore, observing a membrane-embedded reverse turn in an α integrin-TM different from αIIb would support that this structural feature is conserved, and that it represents one of the possible conformations of the GFFKR motif in all integrins.

We have chosen to use the system αLβ2 because of its importance in leukocytes, where it is involved in adhesion, migration, cytotoxicity, proliferation, and antigen presentation [34]. The importance of the GFFKR motif in αL has been demonstrated by looking at the effect of deletion of this motif in a transgenic mouse, which led to a constitutive αLβ2-mediated cell adhesion [35]. Additionally, in these fast-migrating cells, integrin deactivation is particularly important, and we proposed previously that the latter could be facilitated by hydrogen bond interaction between TMs [36]–[38]. Thus, to elucidate if the reverse turn at the GFFKR motif is also found in αL when the peptide is reconstituted in bicelles, and to delineate the αL interfacial residues involved in αL/β2 interaction, we have expressed, purified and determined the structure of αL TM in bicelles by solution NMR. Further, titration experiments with non-labeled β2 TM show which residues in αL are most affected by this interaction.

## Experimental Procedures

### Integrin αL TM and β2 TM Expression and Purification

**Figure 1:**
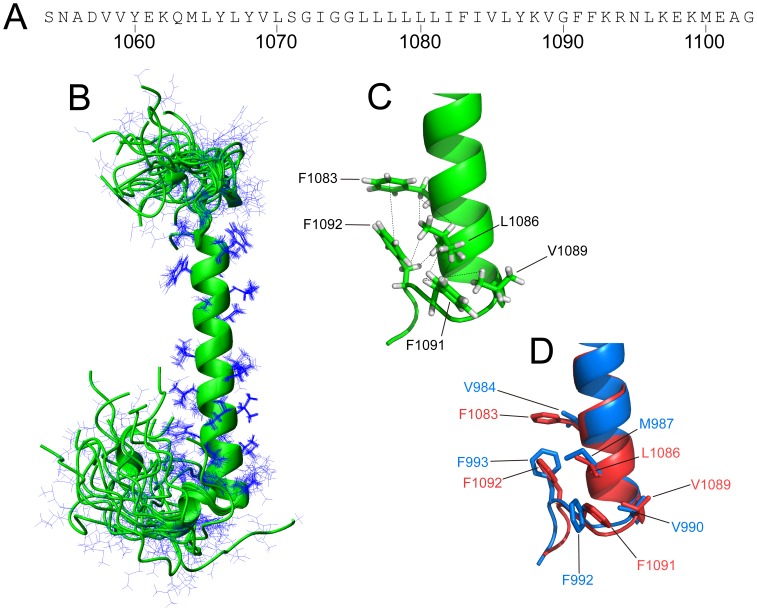
Structure of the integrin αLTM domain. (A) Sequence of the αL TM peptide used in this work with αL chain-context numbering; (B) superposition of an ensemble of 20 calculated simulated annealing structures of αL-TM; side-chains are shown as line representation; (C) αL close up of the IMC region with main residues involved in the formation of the reverse turn. For illustration, some NOEs found are represented as broken lines; (D) structural alignment of αL-TM (Leu-1078 to Arg-1094, in red), and αIIb-TM (PDB code: 2K1A, Leu-979 to Arg-995, in blue). The side-chains are shown as line representation and hydrogen atoms are omitted for simplicity.

The DNA sequence encoding the TM region of human αL integrin, residues 1057–1103 (Fig. 1A), was cloned into a pET-28b expression vector, whereas the sequence for human TM β2 integrin, residues 675–710, was cloned into pNIC28-Bsa4. G685F and G689F mutations at in the GxxxG-like region of β2 TM were subsequently introduced by site-directed mutagenesis with the relevant pair of primers. All constructs were expressed with an N-terminal 6-His tag, cleavable by tobacco etch virus (TEV) protease.

The plasmids were transformed into *E. coli* strain BL21 codon-plus for protein expression. For expression of non-labeled peptides, the culture was grown in terrific broth (TB) media at 37°C until an OD_600_ of 2. Protein expression was induced overnight at 25°C by adding 1 mM IPTG. The cells were harvested by centrifugation at 7500×g and stored at −80°C.

For expression of stable isotope-labeled peptides, the culture was initially grown in LB media at 37°C until an OD_600_ of 0.7. The cells were collected and resuspended into M9 minimal media at 25% of the initial volume, to achieve a high-density culture as described previously [39], [40]. The media was supplemented with ^15^N-NH_4_Cl, ^13^C-glucose, ^13^C/^2^H-glucose, or D_2_O (Cambridge Isotope Laboratories), according to the desired labeling scheme: ^15^N-labeled, ^15^N/^13^C-labeled, and ^15^N/^13^C-labeled with partial (50%) and complete (99%) deuteration. Cultures were further grown for 1 h before inducing protein expression with 1 mM IPTG at 25°C overnight. Cells were harvested as mentioned above and stored at −80°C.

Cell pellets were resuspended in lysis buffer containing 20 mM Tris pH 8.0, 300 mM NaCl, 5 mM imidazole, 2 mM β-mercaptoethanol and 10% glycerol. Complete lysis was achieved by adding 1.5% Triton X-100, followed by sonication and microfluidization. The crude cell lysate was clarified by centrifugation at 40,000×g and applied onto pre-equilibrated Ni-NTA resin (Bio-Rad Profinity IMAC Ni^2+^-charged). The resin was washed with the 30 column volume (CV) of washing buffer A and 3 CV of washing buffer B. Washing buffer A consists of lysis buffer supplemented with 25 mM imidazole while washing buffer B consists of washing buffer A supplemented with 1.8 mM N-dodecyl-β-D-maltopyranoside (DDM). The bound peptide was cleaved on-column by adding TEV protease with 3 hours incubation period at room temperature. Cleaved peptide was collected, TCA-precipitated and lyophilized. The peptides were extracted by adding methanol to the lyophilized powder and removing the precipitates by centrifugation.

Peptide identity and purity was assessed by SDS-PAGE and MALDI-TOF MS (Fig. S1 in File S1). When deemed necessary, the peptides were further purified by using reversed-phase HPLC. Purification was achieved by applying isopropanol-acetonitrile (80/20 (v/v) with 0.1% TFA) linear gradient on a Phenomenex Jupiter C18 semipreparative column (250×10 mm, 300 Å pore size, 5 µm particle size). The peptides were lyophilized and stored in −20°C.

### NMR Sample Preparation

The phospholipid bicelle system was composed of 1,2-dimyristoyl-*sn*-glycero-3-phosphate (DMPC, Avanti Polar Lipids) and 1,2-dihexanoyl-*sn*-glycero-3-phosphate (DHPC, Avanti Polar Lipids). Peptide-DMPC mixture in methanol was dried under N_2_ stream followed by high vacuum. DHPC solution in appropriate buffer was added into the dried peptide-DMPC mixture, vortexed and sonicated. In this way, lyophilized αL TM peptides were reconstituted into DHPC-DMPC bicelle (3% w/v, q = 0.3) at 0.6 mM (corresponding to approximately 1∶100 peptide-to-lipid ratio) buffered with 50 mM potassium phosphate at pH 6.5.

Partial alignment of the αL-TM/bicelle complexes relative to magnetic field was obtained by using compressed and stretched polyacrylamide hydrogels [41], [42]. Axially symmetric alignment tensor coefficients (axiality and rhombicity) were calculated using MODULE [43]. Briefly, 250 µL gels were co-polymerized with 0.6 mM αL TM-bicelle in 50 mM potassium phosphate at pH 6.5, from a 4.2% or 4.6% w/v solution of acrylamide, bis-acrylamide, and 2-acrylamido-2-methyl-1-propanesulfonate (AMPS) with acrylamide-bis ratio of 49∶1 (w/w) and acrylamide-AMPS molar ratio of 94∶6.

*NMR Spectroscopy –* NMR experiments were performed at 30°C using Bruker Avance-III 800 and Avance-II 700 NMR spectrometers with cryogenic probes. Sodium 2,2-dimethyl-2-silapentane-5-sulfonate (DSS) was used as the internal reference for ^1^H nuclei. The chemical shifts of ^13^C and ^15^N nuclei were calculated from the ^1^H chemical shifts. The NMR data were processed using TopSpin 3.1 (www.bruker-biospin.com) and analyzed using CARA (www.nmr.ch). Sequence-specific assignment of backbone ^1^H^N^, ^15^N, ^13^C’ and ^13^C^α^ was achieved by using 2D [^1^H-^15^N]-TROSY-HSQC, 3D TROSY-optimized HNCO, HNCA, HN(CO)CA, HN(CA)CB, and HN(COCA)CB experiments on a fully-deuterated ^15^N/^13^C-labeled αL TM. Side-chain resonances were assigned using 3D ^15^N-resolved NOESY-HSQC (120 ms mixing time), (H)CCH-TOCSY and ^13^C-resolved NOESY-HSQC (120 ms mixing time) on non-deuterated and partially-deuterated ^15^N/^13^C-labeled αL TM. To identify membrane-embedded residues, the NMR sample was lyophilized overnight and reconstituted in 99% D_2_O. Immediately after reconstitution, 2D [^1^H-^15^N]-TROSY-HSQC was collected. The spectra measuring ^1^H-^15^N heteronuclear steady-state NOE were acquired with and without a 3s period of proton saturation. Titration experiments were performed by adding increasing amounts of non-labeled β2 TM to 50 µM ^15^N-labeled αL TM until there were no further changes in the [^1^H-^15^N]-HSQC spectra, from 1∶0.5 to 1∶6 α/β molar ratio.

### Structure Calculation

NOE distance restraints to calculate the structures of αL TM domain were obtained from ^15^N-NOESY-HSQC and ^13^C-NOESY-HSQC spectra, respectively. Backbone dihedral angle restraints (φ and ψ) were derived from ^13^C’, ^13^C^α^, ^13^C^β^, ^1^H^α^ and ^1^H^β^ chemical shift values using TALOS [44]. The short-range and medium range NOE connectivities were used to establish the sequence-specific ^1^H NMR assignment and to identify elements of the regular secondary structure. Structure calculations were performed using CYANA 3.0 [45], [46] and visualized using MOLMOL [47] and PyMOL (Delano Scientific). CNS 1.3 [48], [49] was used to refine the structure using the standard simulated annealing protocol. A total of 20 structures have been calculated and the structure statistics are summarized in Table S1 in File S1. The structure of integrin αL TM domain has been deposited on the Protein Data Bank (PDB) with ID 2m3e, and the assigned chemical shifts have been deposited on the Biological Magnetic Resonance Bank (BMRB) with ID 18958.

## Results

### Structure of the αL TM Domain in Phospholipid Bicelles

The structure of bicelle-reconstituted integrin αL TM domain (αL) was determined by using a set of 2D and 3D NMR experiments on the ^15^N/^13^C-labeled peptide with varying deuteration levels. Upon reconstitution into DHPC-DMPC bicelles, αL forms a linear α-helix which extends approximately from Leu-1065 to Val-1089 (Fig. 1A–B and Fig. S2 in File SS). This α-helix comprises ∼50% (25 residues) of the whole peptide. This is significantly lower than ∼80–90% α-helix estimated by FTIR when this peptide was reconstituted in lipid bilayers (Fig. S3A in File S1), but the value is comparable to ∼60% (30 residues) estimated from CD when the peptide was reconstituted in bicelles (Fig. S3B in File S1).

**Figure 2:**
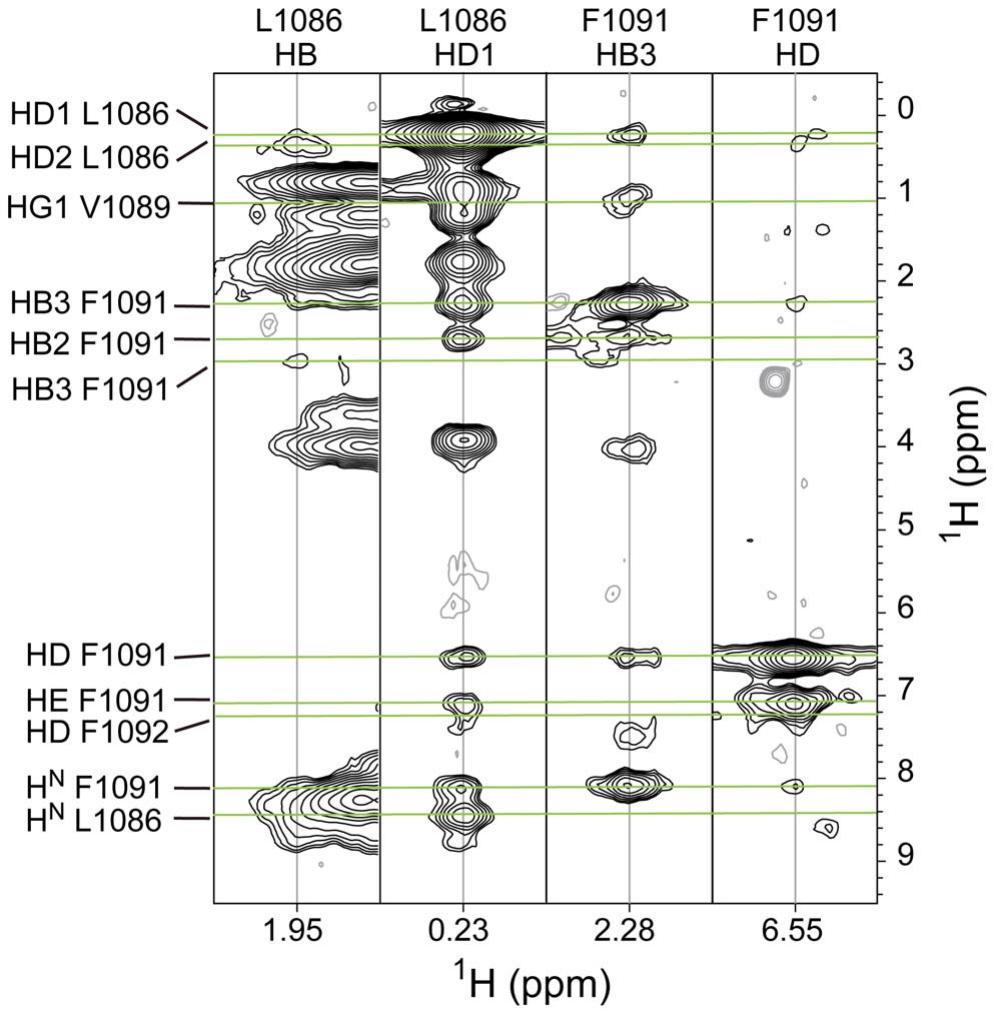
NOE connectivity in the IMC region of αL TM. Four selected strips from ^15^N-NOESY-HSQC and ^13^C-NOESY-HSQC show some NOE contacts among Leu-1086, Val-1089, Phe-1091, and Phe-1092.

The conserved GFFKR motif follows immediately the α-helix, with Phe-1091 and Phe-1092 reinserting into the bicelle (Fig. 1B–C) and forming a backbone reversal, only observed previously in αIIb in bicelles [16]. The structure is consistent with Phe-1091 making hydrophobic contacts to Leu-1086 and Val-1089, whereas the side-chain of Phe-1092 packs against Phe-1083 and Leu-1086, as indicated by NOE connectivities between these residues (Fig. 1C and Fig. 2). Thus Phe-1083, Leu-1086, Phe-1091 and Phe-1092 constitute a hydrophobic surface which potentially forms an interaction site with β2-TM. The superposition of our αL-TM model and that reported for αIIb-TM shows high structural similarity between the two proteins (Fig. 1D). This strongly suggests that the backbone reversal of the GFF motif is a characteristic structural feature of α integrin subunits when reconstituted in bicelles. Alignment of the rest of the TM domains produced an αC RMSD of 0.83 Å. In comparison, the αC RMSD with a recently publised α1 TM structure in micelles for which no reverse turn was observed [24] was 1.3 Å.

**Figure 3:**
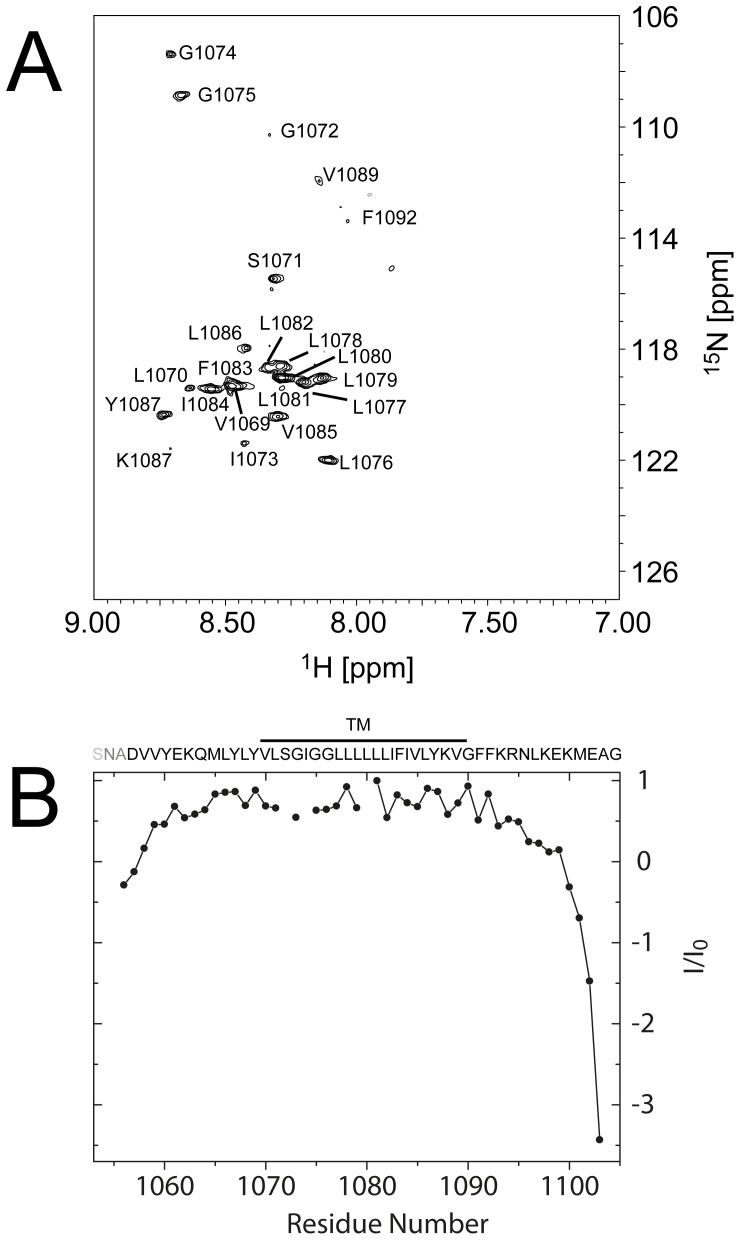
Integrin αL TM H^N^-H_2_O chemical exchange and backbone dynamics. (A) [^1^H-^15^N]-TROSY-HSQC spectra in 99% D_2_O. The cross peaks are labeled by one-letter code and the residue number; (B) [^1^H, ^15^N] steady-state heteronuclear NOE experiment. The three extra N-terminal residues, SNA, are grayed out. The residues protected from H/D exchange are indicated by a line, and labeled as TM.

The membrane-embedded region of αL was identified through an H^N^-D_2_O exchange experiment. From a comparison between [^1^H-^15^N]-TROSY-HSQC spectra in water and in 99% D_2_O, amide protons of a stretch of 21 residues from Val-1069 to Val-1089, plus Phe-1092 in the GFFKR motif, are protected from exchange (Fig. 3A). This number is considerably lower than the 35 exchange-protected residues estimated from FTIR using the peptide reconstituted in lipid bilayers (see IR spectra before and after exchange in Fig. S3C in File S1).

The dynamic properties of αL were studied using a steady-state [^1^H, ^15^N] heteronuclear NOE experiment. The majority of the residues correspond to a well-folded structure, with the central part, Val-1059 to Asn-1095, being the most stable (Fig. 3B). The backbone RMSD of the central TM region from a set of 20 conformers is 0.23 Å, indicating a well-defined structure. On the other hand, the N and C termini, especially the latter, are highly dynamic regions (see also Fig. 1B).

### Interaction between the TMs of αL and β2

**Figure 4:**
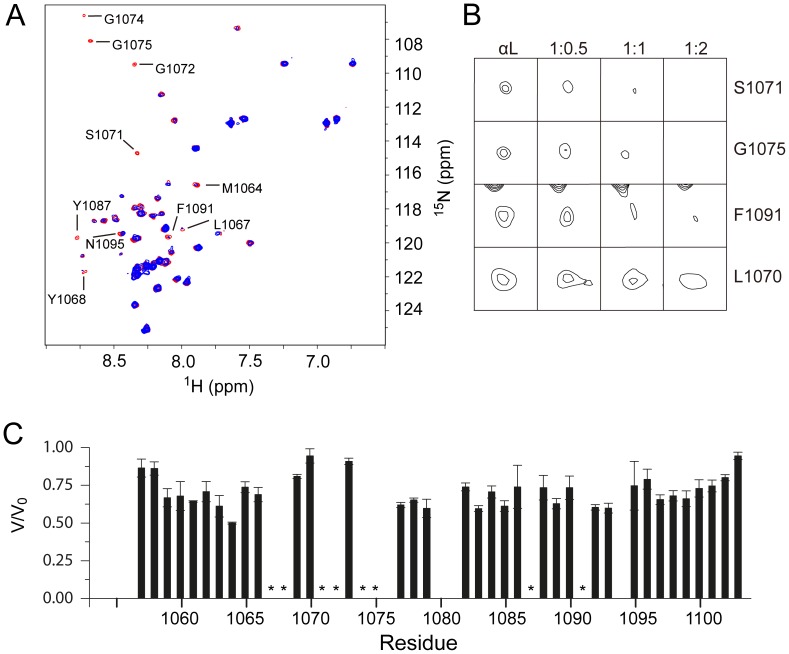
Interaction between αL and β2 TM domains. (A) Result of the titration of ^15^N-labeled αL TM by unlabeled β2 TM at αL/β2 1:2 molar ratio, monitored by [^1^H-^15^N] -HSQC. The spectra in red represent the αL TM alone, while the one in blue was obtained after addition of β2 TM. Residues most affected are labeled; (B) Selected regions in the [^1^H-^15^N] –HSQC spectrum at increasing αL/β2 molar ratios indicated showing intensity reduction and disappearance of selected peaks Ser-1071, Gly-1075, Phe-1091. Leu-1070 is shown for comparison; (C) Peak intensities expressed as ratio between peak volume after (V) and before (V_0_) addition of unlabeled β2 TM to labeled αL TM. Residues that experience extreme line broadening are indicated by *. Missing/overlapping resonances, e.g., R1094, are not shown.

**Figure 5:**
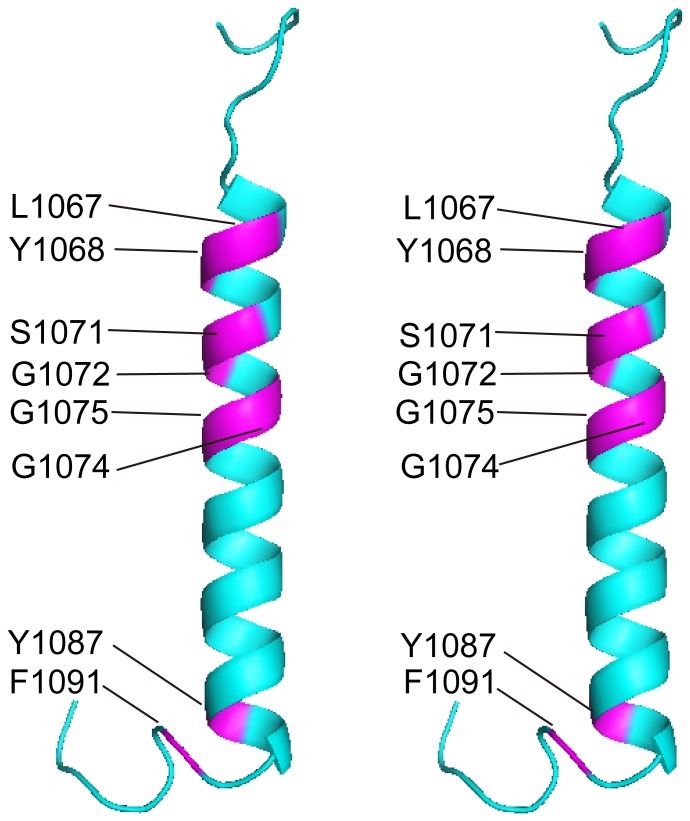
Putative interacting face of αL TM. Stereo view ribbon representation of the αL TM domain (Val-1058 to Lys-1097) with residues most affected by heterodimerization with β2 shown in magenta.

To determine what residues in αL mediate interaction with β2, we titrated unlabeled β2 TM into ^15^N-labeled αLTM, and monitored the changes in the [^1^H-^15^N]-HSQC spectra. Qualitative examination of the spectra at a 1∶2 αL/β2 molar ratio revealed eight residues with pronounced intensity reduction upon addition of β2 peptide, namely Leu-1067, Tyr-1068, Ser-1071, Gly-1072, Gly-1074, Gly-1075, Tyr-1087 and Phe-1091 (Fig. 4A). Met-1064 also showed intensity reduction, albeit less obvious, whereas Asn-1095 experienced a chemical shift change. Intensity of these resonances was reduced with increasing β2 peptide, until the signal disappeared (Fig. 4B). A summary of these changes for the α/β molar ratio 1∶2 is shown in Fig. 4C. The same results were observed when the titration was performed with a β2 TM mutant where the ‘G’ residues in the GxxxG-like motif were changed to Phe, i.e., G685F and G689F (data not shown). The latter confirms that β2 does not interact with αL via its GxxxG-like motif, consistent with the reported αIIb/β3 model of interaction for resting integrins [10], [16], [50]. The affected residues cluster on one side of the αL-TM, with residues Ser-1071 and Gly-1075, located at positions 1 and 5 in the OMC region GxxxG-like motif, having the same orientation as the residues involved in the IMC (Fig. 5). At the same time, these locations are separated by a stretch on non interacting residues, consistent with the TM separation previously observed for the αIIβ/β3 system (see above). Thus, the putative IMC and OMC regions of αL TM are oriented to form a plausible interacting face with β2 TM.

The presence of αβ heterodimerization in our titration is supported by comparison of the TROSY-HSQC spectrum of 0.1 mM αL with that of 0.6 mM concentration obtained in identical conditions shows that increasing αL concentration does not lead to intensity reduction of the peaks (Fig. S4 in File S1). Therefore, the peak intensity reduction observed in our titration experiment is not caused by αL homodimerization. Also, membrane-mimicking screening studies [51] show a predominant presence of αβ heterodimers in a 1∶1 integrin αβ-TM mixture at 0.1 mM, identical concentration to that used here, or even at 0.01 mM. In the latter report, in contrast, is was shown that homomeric species increase in DPC and SDS micelles, and also in organic solvents [51].

## Discussion

### Importance of Environment for Integrin TM Reconstitution

Phospholipid bicelles have been successfully used for structural determination of membrane proteins by solution NMR [52], especially with bicelles of low q-ratios. The inclusion of a small proportion of long-chain lipid makes this system more membrane-like than micelles, yet still reasonably small for solution NMR studies. The better suitability of bicelles, relative to micelles, has been demonstrated in the study of the integrin αIIbβ3 TM heterodimer in bicelles [16], [51]; in micelles, these two TMs were not found to heterodimerize [51], [53].

In addition, the unique backbone reversal for αIIb, was reported in bicelles [17] and full length integrin in biological membranes [15]. In presence of detergent micelles or organic solvents, the region encompassing the GFFKR motif adopted an α-helical conformation in αIIb and several other integrins [18]–[23], [54], even when the TM domain was also present (e.g., [24], [54]). Thus, not only the reconstitution environment may play a critical role for the observation of this reverse turn, but cytoplasmic peptide length and proper conformation of the TM region may be important. Indeed, the reverse turn formed by the GFFKR motif makes contacts with several side-chains in the α TM domain, as we (this paper) and others [16] have observed. Herein a hydrophobic packing is proposed in the αL-TM for the reinserted Phe-1091 and Phe-1092 against Phe-1083 and Leu-1086. The titration data also suggests that Phe-1091 is strongly involved in the interaction with β2 TM, which indicates that the hydrophobic surface formed by Phe-1091 and Phe-1092, Phe-1083 and Leu-1086 is important in the interaction with β2 TM.

We also note that in lipid bilayers (FTIR experiments) 14 more residues were calculated to be protected from H/D exchange relative to the bicelle system (NMR). This higher number is unlikely to only originate from the inevitably larger error incurred when measuring band areas in IR. Rather, these differences must be of experimental origin; in the NMR experiment, bicelles that have a high curvature are re-solubilized in D_2_O. In contrast, in the FTIR experiment, preformed planar lipid bilayers are exposed to D_2_O-saturated air. Whether or not the reverse turn is present in the FTIR experiment is not known, but even considering it is present and completely protected from H/D exchange, this is not sufficient to account for these results. We propose that the extra protection to H/D exchange in membranes is due to the formation of a larger proportion of α-helix in extramembrane domains. Indeed, a higher helical content was observed in the FTIR studies performed in membranes (see above), compared to NMR and CD techniques, where bicelles were used. The increased α-helical content in extramembrane regions in supported lipid bilayers relative to the solution NMR conditions may arise from a far more dehydrated environment in the former. Tight binding of these α-helical domains to the membrane may explain the increased H/D exchange protection. These regions then become unbound, disordered and exposed to solvent when reconstituted in bicelles. The above results suggest that data obtained in organic solvents, micelles or bicelles probably underestimates the helical content and membrane binding behavior of juxtamembrane domains, when compared to lipid bilayers. Indeed, in the present work even the reverse turn formed by the GFFKR motif is not totally protected against H/D exchange in bicelles: Phe-1092 is protected, but Gly-1090 and Phe-1091 are not (Fig. 3A), although as discussed above this may arise from experimental protocol differences. In αIIb-TM, both of the equivalent Phe residues, showed little broadening after exposure to Mn^2+^EDDA2 and H^N^-H_2_O chemical exchange [17], consistent with some degree of protection. Differences in methodology might also account for the shorter membrane-embedded region for αL, relative to αIIb, determined in the present paper.

### Structure of αL in Bicelles

The results of αL structure calculation show that the bicelle-embedded αL peptide adopts a well-defined, highly α-helical conformation at the membrane-spanning region, as expected from a single-pass TM domain. The TM boundaries we found here are generally in agreement with previous studies [17], [54], [55]. The rest of the αL structure is remarkably similar to αIIb, especially with regards to the two proposed interaction points with the β2 chain at the two ends of the TM domain, i.e. the OMC and IMC regions.

In the OMC region, both alternative models proposed for α-β heterodimer interaction predict the small residues in the α TM GxxxG-like motif to face the β TM [11], as observed in the αIIbβ3 interface [16]. In αL, a polar residue, Ser-1071, was hypothesized to form a polar interaction with Thr-686 at the binding partner β2 [36]. Both the alignment of Ser-1071 with Gly-1075 and the hydrophobic IMC region, together with the sensitivity of these residues to titration with the β2 peptide, are consistent with this part of αL forming a polar interaction with β2. As polar interactions are stronger in non-polar environments [56], [57], this has been proposed to have an impact on integrin deactivation, which is important during leukocyte migration [35]. However, further characterization of the αL/β2 interaction is necessary to confirm that point. The OMC region of αL may extend as far as Tyr-1068 and Met-1064, as suggested by the titration experiments.

A chemical shift change was also observed in Asn-1095. This residue was reported to be involved in heterodimerization of the αLβ2 cytoplasmic domain [21]. The equivalent residue in αIIb, Asn-996, experienced a large chemical shift change upon heterodimerization with β3 TM domain [16]. It is possible that the changes observed in αIIbβ3 Asn-996 are indirectly caused by a nearby salt bridge, Arg-995(αIIb)-Asp723(β3) [16]. This salt bridge should also be present in αLβ2, between αL Arg-1094 and β2 Asp-731 [21]. Unfortunately, peak crowding of a large portion of the cytoplasmic tail prevented us from examining the behavior of other residues near this region, e.g. Arg-1094 and Lys-1097.

In summary, we have shown that the conserved GFFKR motif in α integrins forms a structural motif, a backbone reversal, that can be observed when TM domain, cytoplasmic tail and a lipid environment consisting or membranes or bicelles are present, although cytoplasmic tail length may also be an important variable for the stability of this turn [54]. This backbone reversal has been now observed in two different α integrins TMs, αIIb and αL. This is likely to be one of the structural forms of this motif, present in the inactive or intermediate activation state of integrins. We also show that for αL/β2, interaction in the TM domain proper takes place through a remarkably similar structural organization to αIIb/β3, with features reminiscent of OMC and IMC clasps. Further studies will include mutagenesis at the predicted α-β interface to confirm the observed interaction, and crucially, determination of interhelical NOE contacts that can unequivocally describe the precise nature of the polar interaction in αL/β2 pairs. This work is presently underway.

## Supporting Information

File S1 **Includes Table S1, Figures S1–S3.** Table S1. Structure statistics for the selected 20 structures of integrin αL TM domain. **Figure S1. Expression and purification of integrin αL TM and β2 TM peptides.** (A) SDS-PAGE of methanol-extracted (meOH) and HPLC-purified (HPLC) αL-TM and β2-TM peptides. (B) MALDI-TOF MS spectra of the HPLC fractions corresponding to αL TM and β2 TM peptides. The letters inside square brackets identify the charged species forming a particular peak: M, H, Na, K corresponds to peptide, proton, sodium ion, and potassium ions, respectively. The charge of each species is indicated outside the square bracket. **Figure S2. Secondary structure of the peptide αL used, based on NOE connectivity.** Sequential and medium-ranged NOE connectivity between residues are displayed as bands under the respective residues. The d_αN_(i,i+4) connectivities indicate the α-helical structure, extending from Leu-1065 to Val-1088. **Figure S3. Secondary structure of integrin TM peptides.** (A) FT-IR spectra of integrin TM α (upper) and β (lower) peptides reconstituted in DMPC lipid bilayers indicating that both TM peptides are almost completely α-helical. The amide I peak centered at 1657 cm^−1^ (blue) and the Fourier self-deconvolved spectra (red) are shown; (B) CD spectra of integrin TM peptides in DHPC-DMPC bicelles, showing minima at 209 and 222 nm, indicating a high proportion of α-helical form for both peptides; (C) Amide I and amide II regions of integrin TM peptides when bulk water was removed (red) and after D_2_O-saturated air exposure (blue). The decrease in the amide II area at ∼1550 cm^−1^ indicates H-D exchange. **Figure S4. Effect of increasing concentration on the [^1^H-^15^N]-TROSY-HSQC spectrum of αL-TM.** αL TM peptide at 0.1 mM (A) or 0.6 mM (B). αL was reconstituted into DHPC-DMPC bicelles (3% w/v, q = 0.3) buffered with 50 mM potassium phosphate at pH 6.5 and spectra were recorded at 305 K.
(DOCX)

## References

[1] Hemler M (1999) Extracellular Matrix, Anchor, and Adhesion Proteins; Kreis T, and Vale, R., editor. Oxford: Oxford University Press.

[2] Carman CV, Springer TA (2003) Integrin avidity regulation: are changes in affinity and conformation underemphasized? Curr Opin Cell Biol 15: 547–556. 14519389 10.1016/j.ceb.2003.08.003

[3] Watt FM (2002) Role of integrins in regulating epidermal adhesion, growth and differentiation. EMBO J 21: 3919–3926. 12145193 10.1093/emboj/cdf399 PMC126145

[4] Kim M, Carman CV, Springer TA (2003) Bidirectional transmembrane signaling by cytoplasmic domain separation in integrins. Science 301: 1720–1725. 14500982 10.1126/science.1084174

[5] Hantgan RR, Paumi C, Rocco M, Weisel JW (1999) Effects of ligand-mimetic peptides Arg-Gly-Asp-X (X = Phe, Trp, Ser) on αIIb β3 integrin conformation and oligomerization. Biochemistry 38: 14461–14474. 10545168 10.1021/bi9907680

[6] Travis MA, Humphries JD, Humphries MJ (2003) An unraveling tale of how integrins are activated from within. Trends Pharmacol Sci 24: 192–197. 12707006 10.1016/S0165-6147(03)00069-5

[7] Lu CF, Takagi J, Springer TA (2001) Association of the membrane proximal regions of the α and β subunit cytoplasmic domains constrains an integrin in the inactive state. J Biol Chem 276: 14642–14648. 11279101 10.1074/jbc.M100600200

[8] O’Toole TE, Katagiri Y, Faull RJ, Peter K, Tamura R, et al (1994) Integrin cytoplasmic domains mediate inside-out signal transduction. J Cell Biol 124: 1047–1059. 7510712 10.1083/jcb.124.6.1047 PMC2119979

[9] Lu CF, Springer TA (1997) The alpha subunit cytoplasmic domain regulates the assembly and adhesiveness of integrin lymphocyte function-associated antigen-1. J Immunol 159: 268–278. 9200463

[10] Luo BH, Springer TA, Takagi J (2004) A specific interface between integrin transmembrane helices and affinity for ligand. PLoS Biol 2: 776–786. 10.1371/journal.pbio.0020153 PMC423134 15208712

[11] Gottschalk KE, Adams PD, Brunger AT, Kessler H (2002) Transmembrane signal transduction of the αIIbβ3 integrin. Protein Sci 11: 1800–1812. 12070332 10.1110/ps.4120102 PMC2373644

[12] MacKenzie KR, Prestegard JH, Engelman DM (1997) A transmembrane helix dimer: Structure and implications. Science 276: 131–133. 9082985 10.1126/science.276.5309.131

[13] Adair BD, Yeager M (2002) Three-dimensional model of the human platelet integrin aIIbb3 based on electron cryomicroscopy and x-ray crystallography. Proc Nat Acad Sci USA 99: 14059–14064. 12388784 10.1073/pnas.212498199 PMC137836

[14] Partridge AW, Liu S, Kim S, Bowie JU, Ginsberg MH (2005) Transmembrane domain helix packing stabilizes integrin alphaIIbbeta3 in the low affinity state. J Biol Chem 280: 7294–7300. 15591321 10.1074/jbc.M412701200

[15] Zhu J, Luo BH, Barth P, Schonbrun J, Baker D, et al (2009) The structure of a receptor with two associating transmembrane domains on the cell surface: integrin alphaIIbbeta3. Mol Cell 34: 234–249. 19394300 10.1016/j.molcel.2009.02.022 PMC2694939

[16] Lau TL, Kim C, Ginsberg MH, Ulmer TS (2009) The structure of the integrin alphaIIbbeta3 transmembrane complex explains integrin transmembrane signalling. EMBO J 28: 1351–1361. 19279667 10.1038/emboj.2009.63 PMC2683045

[17] Lau TL, Dua V, Ulmer TS (2008) Structure of the integrin alphaIIb transmembrane segment. J Biol Chem 283: 16162–16168. 18417472 10.1074/jbc.M801748200 PMC3259656

[18] Chua GL, Patra AT, Tan SM, Bhattacharjya S (2013) NMR Structure of Integrin alpha4 Cytosolic Tail and Its Interactions with Paxillin. PLoS ONE 8: e55184. 23383101 10.1371/journal.pone.0055184 PMC3561355

[19] Chua GL, Tang XY, Patra AT, Tan SM, Bhattacharjya S (2012) Structure and binding interface of the cytosolic tails of alphaXbeta2 integrin. PLoS ONE 7: e41924. 22844534 10.1371/journal.pone.0041924 PMC3406025

[20] Chua GL, Tang XY, Amalraj M, Tan SM, Bhattacharjya S (2011) Structures and interaction analyses of integrin alphaMbeta2 cytoplasmic tails. J Biol Chem 286: 43842–43854. 22052909 10.1074/jbc.M111.280164 PMC3243538

[21] Bhunia A, Tang XY, Mohanram H, Tan SM, Bhattacharjya S (2009) NMR solution conformations and interactions of integrin alphaLbeta2 cytoplasmic tails. J Biol Chem 284: 3873–3884. 19073598 10.1074/jbc.M807236200

[22] Vinogradova O, Velyvis A, Velyviene A, Hu B, Haas TA, et al (2002) A structural mechanism of integrin alpha(IIb)beta(3) “inside-out” activation as regulated by its cytoplasmic face. Cell 110: 587–597. 12230976 10.1016/s0092-8674(02)00906-6

[23] Vinogradova O, Haas T, Plow EF, Qin J (2000) A structural basis for integrin activation by the cytoplasmic tail of the alpha(IIb)-subunit. Proc Nat Acad Sci USA 97: 1450–1455. 10677482 10.1073/pnas.040548197 PMC26454

[24] Lai C, Liu X, Tian C, Wu F (2013) Integrin α1 has a long helix, extending from the transmembrane region to the cytoplasmic tail in detergent micelles. PLOS ONe 8: e62954 doi:10.1371/journal.pone.0062954 23646163 10.1371/journal.pone.0062954 PMC3639902

[25] Gushiken FC, Patel V, Liu Y, Pradhan S, Bergeron AL, et al (2008) Protein Phosphatase 2A Negatively Regulates Integrin αIIbβ3 Signaling. J Biol Chem 283: 12862–12869. 18334487 10.1074/jbc.M708804200 PMC2442329

[26] Alahari SK, Lee JW, Juliano RL (2000) Nischarin, a Novel Protein That Interacts with the Integrin α5 Subunit and Inhibits Cell Migration. J Cell Biol 151: 1141–1154. 11121431 10.1083/jcb.151.6.1141 PMC2190593

[27] Alahari SK, Nasrallah H (2004) A membrane proximal region of the integrin alpha5 subunit is important for its interaction with nischarin. Biochem J 377: 449–457. 14535848 10.1042/BJ20030411 PMC1223876

[28] Armulik A, Nilsson I, von Heijne G, Johansson S (1999) Determination of the border between the transmembrane and cytoplasmic domains of human integrin subunits. J Biol Chem 274: 37030–37034. 10601259 10.1074/jbc.274.52.37030

[29] Naik UP, Patel PM, Parise LV (1997) Identification of a Novel Calcium-binding Protein That Interacts with the Integrin αIIb Cytoplasmic Domain. J Biol Chem 272: 4651–4654. 9030514 10.1074/jbc.272.8.4651

[30] Tsuboi S (2002) Calcium Integrin-binding Protein Activates Platelet Integrin αIIbβ3. J Biol Chem 277: 1919–1923. 11704681 10.1074/jbc.M110643200

[31] Vallar L, Melchior C, Plançon S, Drobecq H, Lippens G, et al (1999) Divalent Cations Differentially Regulate Integrin αIIb Cytoplasmic Tail Binding to β3 and to Calcium- and Integrin-binding Protein. J Biol Chem 274: 17257–17266. 10358085 10.1074/jbc.274.24.17257

[32] Rantala JK, Pouwels J, Pellinen T, Veltel S, Laasola P, et al (2011) SHARPIN is an endogenous inhibitor of [beta]1-integrin activation. Nat Cell Biol 13: 1315–1324. 21947080 10.1038/ncb2340 PMC3257806

[33] Nevo J, Mai A, Tuomi S, Pellinen T, Pentikainen OT, et al (2010) Mammary-derived growth inhibitor (MDGI) interacts with integrin [alpha]-subunits and suppresses integrin activity and invasion. Oncogene 29: 6452–6463. 20802519 10.1038/onc.2010.376

[34] Kinashi T (2007) Integrin regulation of lymphocyte trafficking: lessons from structural and signaling studies. Adv Immunol 93: 185–227. 17383542 10.1016/S0065-2776(06)93005-3

[35] Semmrich M, Smith A, Feterowski C, Beer S, Engelhardt B, et al (2005) Importance of integrin LFA-1 deactivation for the generation of immune responses. J Exp Med 201: 1987–1998. 15955836 10.1084/jem.20041850 PMC2212031

[36] Vararattanavech A, Chng CP, Parthasarathy K, Tang XY, Torres J, et al (2010) A transmembrane polar interaction is involved in the functional regulation of integrin αLβ2. J Mol Biol 398: 569–583. 20338181 10.1016/j.jmb.2010.03.027

[37] Vararattanavech A, Lin X, Torres J, Tan SM (2009) Disruption of the integrin alphaLbeta2 transmembrane domain interface by beta2 Thr-686 mutation activates alphaLbeta2 and promotes micro-clustering of the alphaL subunits. J Biol Chem 284: 3239–3249. 19029120 10.1074/jbc.M802782200

[38] Chng CP, Tan SM (2011) Proteins Struct Funct Bioinf. 79: 2203–2213. 10.1002/prot.23044 21557324

[39] Sivashanmugam A, Murray V, Cui CX, Zhang YH, Wang JJ, et al (2009) Practical protocols for production of very high yields of recombinant proteins using Escherichia coli. Protein Sci 18: 936–948. 19384993 10.1002/pro.102 PMC2771296

[40] Studier FW (2005) Protein production by auto-induction in high-density shaking cultures. Prot Exp Purif 41: 207–234. 10.1016/j.pep.2005.01.016 15915565

[41] Tycko R, Blanco FJ, Ishii Y (2000) Alignment of Biopolymers in Strained Gels: A New Way To Create Detectable Dipole−Dipole Couplings in High-Resolution Biomolecular NMR. J Am Chem Soc 122: 9340–9341.

[42] Ulmer TS, Ramirez BE, Delaglio F, Bax A (2003) Evaluation of backbone proton positions and dynamics in a small protein by liquid crystal NMR spectroscopy. J Am Chem Soc 125: 9179–9191. 15369375 10.1021/ja0350684

[43] Dosset P, Hus JC, Marion D, Blackledge M (2001) A novel interactive tool for rigid-body modeling of multi-domain macromolecules using residual dipolar couplings. J Biomol NMR 20: 223–231. 11519746 10.1023/a:1011206132740

[44] Cornilescu G, Delaglio F, Bax A (1999) Protein backbone angle restraints from searching a database for chemical shift and sequence homology. J Biomol NMR 13: 289–302. 10212987 10.1023/a:1008392405740

[45] Guntert P, Mumenthaler C, Wuthrich K (1997) Torsion angle dynamics for NMR structure calculation with the new program DYANA. J Mol Biol 273: 283–298. 9367762 10.1006/jmbi.1997.1284

[46] Herrmann T, Guntert P, Wuthrich K (2002) Protein NMR structure determination with automated NOE assignment using the new software CANDID and the torsion angle dynamics algorithm DYANA. J Mol Biol 319: 209–227. 12051947 10.1016/s0022-2836(02)00241-3

[47] Koradi R, Billeter M, Wuthrich K (1996) MOLMOL: a program for display and analysis of macromolecular structures. J Mol Graph 14: 51–55, 29–32. 10.1016/0263-7855(96)00009-4 8744573

[48] Brunger AT (2007) Version 1.2 of the Crystallography and NMR system. Nat Prot 2: 2728–2733. 10.1038/nprot.2007.406 18007608

[49] Brunger AT, Adams PD, Clore GM, DeLano WL, Gros P, et al (1998) Crystallography & NMR system: A new software suite for macromolecular structure determination. Acta Crystallogr, Sect D: Biol Crystallogr 54: 905–921. 9757107 10.1107/s0907444998003254

[50] Berger BW, Kulp DW, Span LM, DeGrado JL, Billings PC, et al (2010) Consensus motif for integrin transmembrane helix association. Proc Nat Acad Sci USA 107: 703–708. 20080739 10.1073/pnas.0910873107 PMC2818961

[51] Suk J-E, Situ AJ, Ulmer TS (2012) Construction of Covalent Membrane Protein Complexes and High-Throughput Selection of Membrane Mimics. J Am Chem Soc 134: 9030–9033. 22626249 10.1021/ja304247f PMC3415561

[52] Vold RR, Prosser RS, Deese AJ (1997) Isotropic solutions of phospholipid bicelles: a new membrane mimetic for high-resolution NMR studies of polypeptides. J Biomol NMR 9: 329–335. 9229505 10.1023/a:1018643312309

[53] Li R, Babu CR, Lear JD, Wand AJ, Bennett JS, et al (2001) Oligomerization of the integrin αIIbβ3: roles of the transmembrane and cytoplasmic domains. Proc Nat Acad Sci USA 98: 12462–12467. 11606749 10.1073/pnas.221463098 PMC60076

[54] Yang J, Ma Y-Q, Page RC, Misra S, Plow EF, et al (2009) Proc Nat Acad Sci USA. 106: 17729–17734. 10.1073/pnas.0909589106 PMC2764936 19805198

[55] Armulik A, Nilsson I, von Heijne G, Johansson S (1999) Determination of the border between the transmembrane and cytoplasmic domains of human integrin subunits. J Biol Chem 274: 37030–37034. 10601259 10.1074/jbc.274.52.37030

[56] Zhou FX, Merianos HJ, Brunger AT, Engelman DM (2001) Polar residues drive association of polyleucine transmembrane helices. Proc Nat Acad Sci USA 98: 2250–2255. 11226225 10.1073/pnas.041593698 PMC30124

[57] Zhou FX, Cocco MJ, Russ WP, Brunger AT, Engelman DM (2000) Interhelical hydrogen bonding drives strong interactions in membrane proteins. Nat Struct Biol 7: 154–160. 10655619 10.1038/72430

