# Supplementary material for: Transmembrane and Juxtamembrane Structure of αL Integrin in Bicelles
Source: PLoS One. 2013 Sep 12;8(9):e74281. doi: 10.1371/journal.pone.0074281 (PMC3771934; doi:10.1371/journal.pone.0074281)
Supplement: File S1 — Includes Table S1, Figures S1–S3. Table S1. Structure statistics for the selected 20 structures of integrin αL TM domain. Figure S1. Expression and purification of integrin αL TM and β2 TM peptides. (A) SDS-PAGE of methanol-extracted (meOH) and HPLC-purified (HPLC) αL-TM and β2-TM peptides. (B) MALDI-TOF MS spectra of the HPLC fractions corresponding to αL TM and β2 TM peptides. The letters inside square brackets identify the charged species forming a particular peak: M, H, Na, K corresponds to peptide, proton, sodium ion, and potassium ions, respectively. The charge of each species is indicated outside the square bracket. Figure S2. Secondary structure of the peptide αL used, based on NOE connectivity. Sequential and medium-ranged NOE connectivity between residues are displayed as bands under the respective residues. The dαN(i,i+4) connectivities indicate the α-helical structure, extending from Leu-1065 to Val-1088. Figure S3. Secondary structure of integrin TM peptides. (A) FT-IR spectra of integrin TM α (upper) and β (lower) peptides reconstituted in DMPC lipid bilayers indicating that both TM peptides are almost completely α-helical. The amide I peak centered at 1657 cm−1 (blue) and the Fourier self-deconvolved spectra (red) are shown; (B) CD spectra of integrin TM peptides in DHPC-DMPC bicelles, showing minima at 209 and 222 nm, indicating a high proportion of α-helical form for both peptides; (C) Amide I and amide II regions of integrin TM peptides when bulk water was removed (red) and after D2O-saturated air exposure (blue). The decrease in the amide II area at ∼1550 cm−1 indicates H-D exchange. Figure S4. Effect of increasing concentration on the [1H-15N]-TROSY-HSQC spectrum of αL-TM. αL TM peptide at 0.1 mM (A) or 0.6 mM (B). αL was reconstituted into DHPC-DMPC bicelles (3% w/v, q = 0.3) buffered with 50 mM potassium phosphate at pH 6.5 and spectra were recorded at 305 K. (DOCX) [file pone.0074281.s001.docx]

**SUPPLEMENTAL DATA**

Table 1. Structure statistics for the selected 20 structures of integrin αL TM domain

| NMR restraints | | |
| --- | --- | --- |
|  | Total unambiguous distance restraints | 600 |
|  | Intra residual | 253 |
|  | Sequential ( \| i – j \| = 1) | 155 |
|  | Short-range ( \| i – j \| <=1) | 408 |
|  | Medium ( 2 ≤ \| i – j \| ≤ 4) | 168 |
|  | Long range ( \| i – j \| ≥ 5) | 24 |
| Dihedral angle restraints | | 46 |
| Hydrogen bond restraints^a^ | | 20 |
| RDC restraints | | 47 |
| RMSD from the experimental residual dipolar couplings (Hz) | | |
| ^1^D_NH_ | | 0.44 ± 0.03 |
| RMSD from the average atomic coordinates (residues 1065-1093, Å)^b^ | | |
|  | Backbone atoms | 0.23 ± 0.05 |
|  | All heavy atoms | 0.65 ± 0.07 |
| Ramachandran analysis (%) | | |
|  | Residues in most favored regions | 75.7 |
|  | Residues in additional allowed regions | 24.1 |
|  | Residues in generously allowed regions | 0.2 |
|  | Residues in disallowed regions | 0.0 |

^a^Backbone hydrogen bonds of α-helix are applied for regions confirmed to be α-helix according to local NOE pattern and H^N^-H_2_O chemical exchange experiment.

^b^Statistics are calculated and averaged over an ensemble of the 20 structures with lowest target function from CYANA.

**
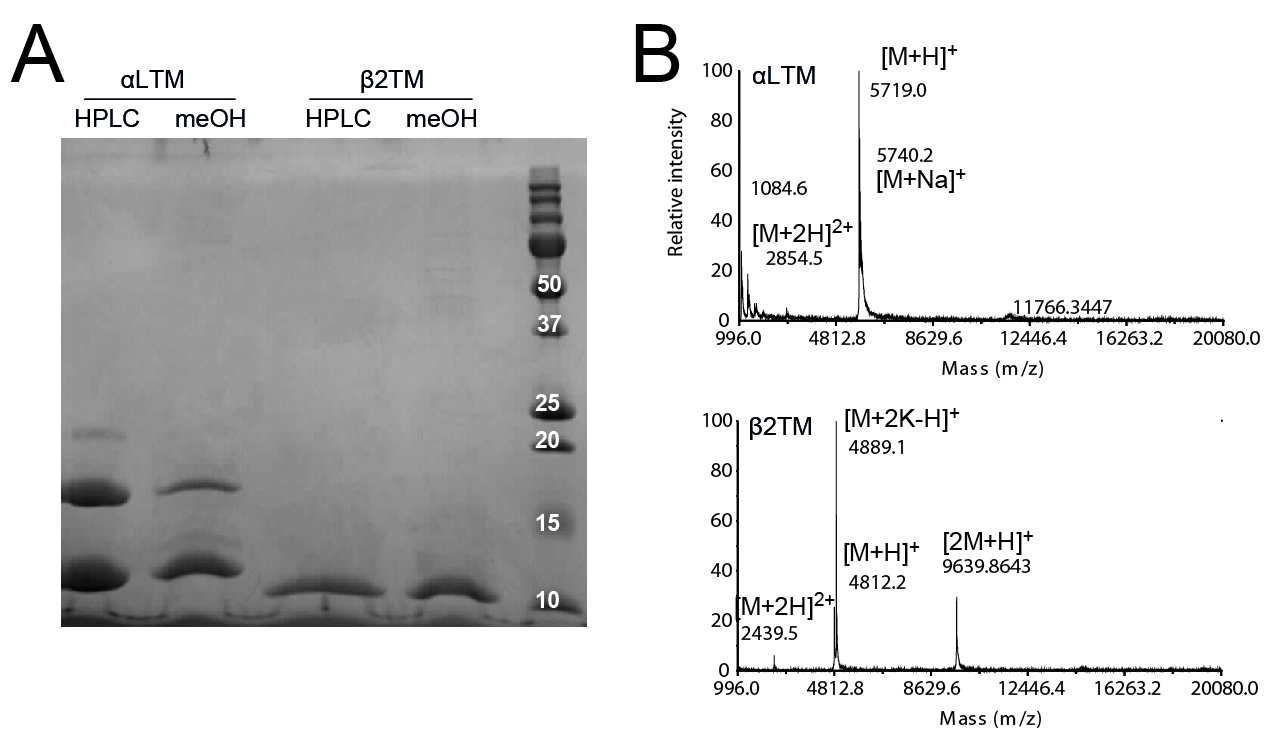
**

**Figure S1. Expression and purification of integrin αL TM and β2 TM peptides.** (A) SDS-PAGE of methanol-extracted (meOH) and HPLC-purified (HPLC) αL-TM and β2-TM peptides. (B) MALDI-TOF MS spectra of the HPLC fractions corresponding to αL TM and β2 TM peptides. The letters inside square brackets identify the charged species forming a particular peak: M, H, Na, K corresponds to peptide, proton, sodium ion, and potassium ions, respectively. The charge of each species is indicated outside the square bracket.


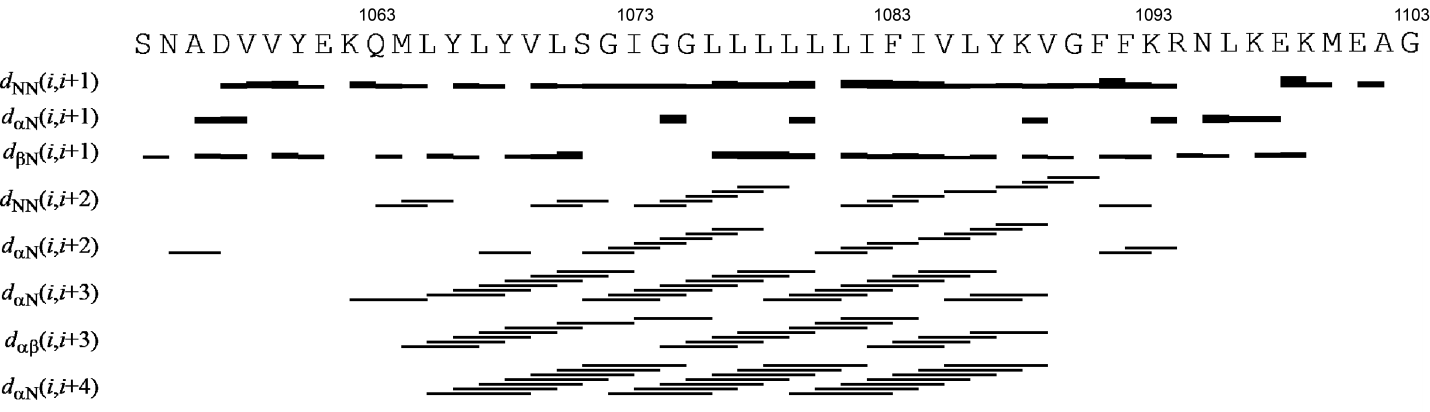


**Figure S2. Secondary structure of the peptide αL used, based on NOE connectivity.** Sequential and medium-ranged NOE connectivity between residues are displayed as bands under the respective residues. The d_αN_(i,i+4) connectivities indicate the α-helical structure, extending from Leu-1065 to Val-1088.


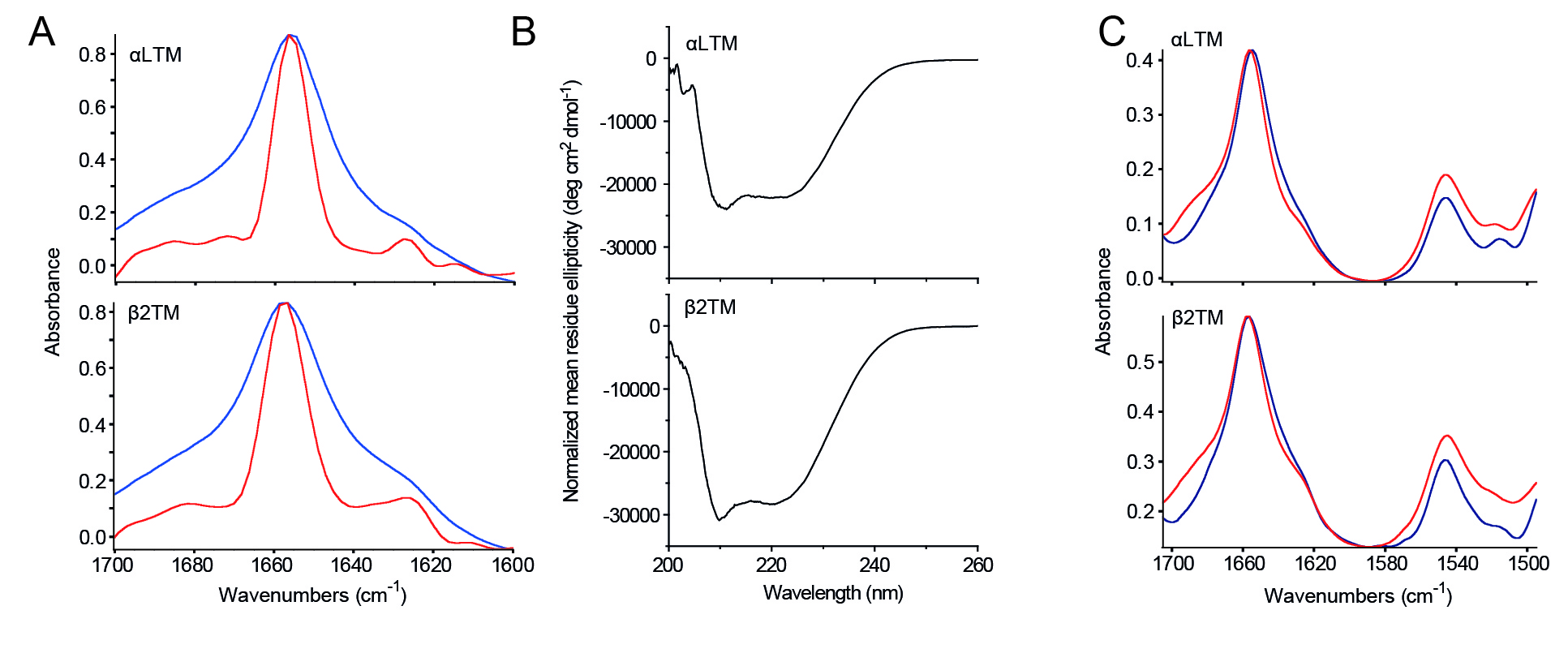


**Figure S3. Secondary structure of integrin TM peptides.** (A) FT-IR spectra of integrin TM α (upper) and β (lower) peptides reconstituted in DMPC lipid bilayers indicating that both TM peptides are almost completely α-helical. The amide I peak centered at 1657 cm^-1^ (blue) and the Fourier self-deconvolved spectra (red) are shown; (B) CD spectra of integrin TM peptides in DHPC-DMPC bicelles, showing minima at 209 and 222 nm, indicating a high proportion of α-helical form for both peptides; (C) Amide I and amide II regions of integrin TM peptides when bulk water was removed (red) and after D_2_O-saturated air exposure (blue). The decrease in the amide II area at ~ 1550 cm^-1^indicates H-D exchange.

**
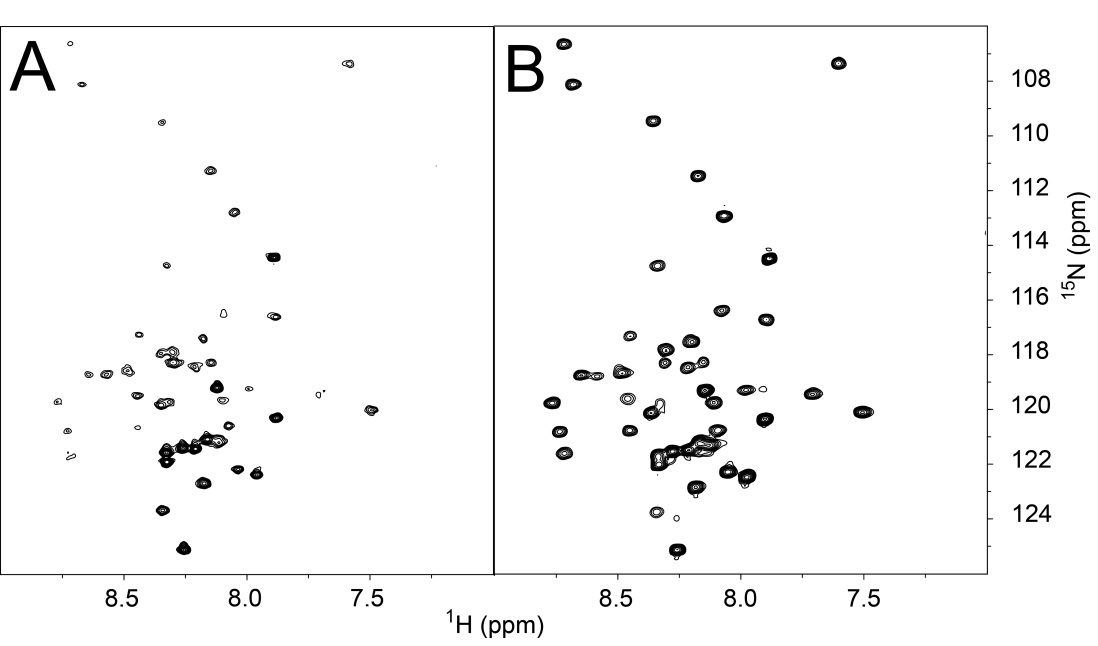
**

**Figure S4. Effect of increasing concentration on the [^1^H-^15^N]-TROSY-HSQC spectrum of αL-TM.** αL TM peptide at 0.1 mM (A) or 0.6 mM (B). αL was reconstituted into DHPC-DMPC bicelles (3% w/v, q = 0.3) buffered with 50 mM potassium phosphate at pH 6.5 and spectra were recorded at 305 K.

**Fourier Transform-Infrared (FT-IR) Spectroscopy.** Infrared spectra were collected on a Nicolet Nexus 470 FT-IR spectrometer purged with dry, CO_2_-free air and equipped with a MCT/A detector cooled in liquid N_2_. Attenuated total reflection (ATR) spectra were measured with a 25-reflection ATR accessory and a wire grid polarizer (Graseby Specac).

*Sample preparation.*Peptide-lipid (dimyristoyl phospatidyl choline; DMPC, Avanti Polar Lipids) mixture at a 1:50 molar ratio was resuspended in water, vortexed and freeze-thawed. The suspension was subsequently dried on a Ge internal reflection element, which was assembled onto the ATR cell. Dry or D_2_O-saturated N_2_ stream was passed through the ATR cell for at least 1 h before collecting the spectra of dry and D_2_O-exchanged samples, respectively.

*Spectral manipulation.* Amide proton H/D exchange was measured from the reduction of amide II band intensity in the non-polarized spectra, which was calculated from the parallel (║) and perpendicular (┴) ATR polarized spectra using the formula 1(║) + 1.44(┴)([1](#_ENREF_1)). The percentage of H/D exchange was calculated from the area of amide II band (A_II_) relative to the area of amide I band (A_I_) in D_2_O-exchanged sample as compared to the dry sample (Eq. 1).


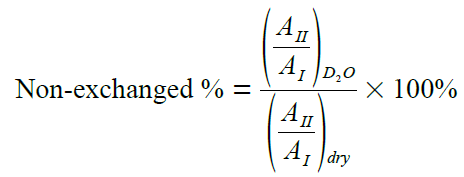
(Equation 1)

To determine the percentage of secondary structure elements, Fourier self-deconvolution was performed on the amide I band. This analysis reveals that almost 100% of the peptide is α-helical, with a maximum centered at 1656 cm^-1^ (Fig. S3A). The dichroic ratio of the amide A band was calculated comparing the spectra obtained in samples exposed to D_2_O with either parallel (║) or perpendicular (┴) polarization.

*Results.* The dichroic ratios for the amide A band of αL TM and β2 TM peptides were 3.6 and 2.7, respectively, indicating that the αL TM helix axis is almost perpendicular respect to the lipid bilayer plane, while the β2 TM helix is more tilted.

Hydrogen/deuterium (H/D) exchange experiments(Fig. S3C) revealed that 70% (35 aa) and 62% (28 aa) of the residues in αL TM and β2 TM peptides, respectively, are protected from exchange. The number is slightly higher than expected for αL TM, as the TM region only consists of ~25 residues. Nevertheless, it indicates that the peptide is embedded in the lipid bilayer. Thus, the FT-IR data show that the integrin TM peptides refold into α-helical TM domain following reconstitution of the lyophilized peptides into lipid bilayers.

**Circular Dichroism.** CD data was collected on Chirascan CD Spectrometer (Applied Photophysics) using a 1 mm quartz cuvette (Hellma). Lyophilized αL TM peptides were reconstituted at 0.5 mg/ml in 3% phospholipid bicelle solution (q = 0.3) buffered with 50 mM potassium phosphate, pH 6.5. CD spectra were acquired from 180 to 260 nm with 1 nm spectral bandwidth and 3 replicates per spectra. Following baseline subtraction, CD data were analyzed in Dichroweb ([2](#_ENREF_2)) by using the CDSSTR method ([3](#_ENREF_3)) and the SMP180 reference set ([4](#_ENREF_4)). CD spectra of both TMs displays typical α-helical peptide and data fitting by using CDSSTR method resulted in ~60% α-helical content for αL TM and β2 TM (Fig. S3B).

1. Marsh, D. (1999) *Biophys. J.***77**, 2630-2637

2. Whitmore, L., and Wallace, B. A. (2004) *Nucleic Acids Res.***32**, W668-W673

3. Compton, L. A., and Johnson, W. C. (1986) *Anal. Biochem.***155**, 155-167

4. Abdul-Gader, A., Miles, A. J., and Wallace, B. A. (2011) *Bioinformatics***27**, 1630-1636
